# Abstracts and Gleanings

**Published:** 1886-12-20

**Authors:** 


### ABSTRACTS AND GLEANINGS.

**Retained Placenta.**—Dr. Hendrick, in a paper read before the New York Medical Association, remarks:

We wish to give our experience, which has been that of the ordinary village and country practitioner, with an obstetric business equal to the average for one-third of a century; and during the past one-quarter of a century we do not recall a case, in our own practice, of the third stage of labor, at full term, exceeding twenty minutes.—unless the time was necessarily consumed in some extra care for the child—and *never* a case of fatal hemorrhage; generally the time occupied from birth of the child to delivery of the placenta has been within ten minutes.

Our custom has been to proceed, without essential delay, by expression, with gentle, guiding traction. Quoting again Playfair, in his advocacy of the Crede method—"that in nineteen cases out of twenty, *without touching the cord*, the bugbear of retained placenta will cease to be a source of dread."

We will add that the same will be true in ninety-nine cases out of the one hundred—and many times better still—by what we may call the Crede and Cazeaux methods combined, which we have essentially practiced for many years. We will give the procedure in detail. We prefer to have the placental end of the cut cord ligated, to retain all the blood, and which may aid in the separation of the placenta. Immediately after tying and severing the cord and due attention to the child, with the woman lying upon her back, wind the cord about one hand and third and fourth fingers—or fingers alone, if the cord be very short—until the hand is brought in close contact with the vulva; the first and second fingers are carried along the upper surface of the cord to the placenta or os uteri, thereby one hand serving all the purposes of two, as illustrated in the traction method of Cazeaux; the other hand over the uterine tumor, its ulnar side defllected, with the fingers carried as well back and around the uterus as possible, while the thumb and thenar eminence serve to antagonize and secure the most efficient possible and uniform grasp of the tumor.

By this time, usually, the womb is ready to respond; and when it responds the grasp is tightened, with a pressure bearing the uterus in the direction of the axis of the superior strait, meanwhile giving direction and slight traction by the cord. At the same time the patient is directed to "bear down," or, what is better, to place the palm of her hand tightly over the mouth and blow against it with all her might, and with it is sure to come the straining effort; and here let it be said and emphasized that pressure by inflation of the lungs—forced inflation—is worth more as an adjuvant expulsive force than all others combined. The leading feature of all, however, is the peculiar, expressive grasp of the uterine tumor, which perhaps cannot be fully told, but must be comprehended by the physician himself—a sort of tactile sense that assures him he is aiding the uterus in its contraction and expulsion of its contents — a sort of peristaltic squeeze, the position of hand aiding both in excitement and direct expulsion. Usually the work is completed with the first uterine pain, which is greatly encouraged and prolonged by the blowing efforts of the patient. Labor is over and the "bugbear of retention" gone by; but the grasp of the uterus, to prevent hemorrhage, is still maintained, without interruption, for some minutes — longer or shorter according to the tone of contraction; and the dose of ergot at this time is a source of safety. Generally, I would not rely upon it as an aid in the third stage of labor; nor do I give the full dose in the close of the second stage to secure contraction of the womb, as is the custom of some, unless measurably sure of completing the third stage ahead of its specific effect, lest it should bring on spasmodic contraction of the womb, which is one of the fruitful sources of retention of placenta.

One thing should have been mentioned, that is respecting the varied positions of the placenta assumed in the different modes of delivery. In the traction method the root of the cord and fœtal surface appear first; by expression alone generally the maternal surface is expelled first; in natural or unaided expulsion it usually descends by one edge, occupying less space in the os uteri and less room laterally in the vagina. The latter or natural position of the placenta in its descent is quite generally maintained, and nearly always when well balanced aid by expression and traction is combined. Its naturalness recommends it; its success is its advocate.

If with well-directed efforts, by whatever method, the after-birth is not delivered, when shall we call it retained placenta? and what shall be done? Authors seem to make the time from half an hour to an hour and a half. We believe the latter time long enough to wait. Our experience is confined only to a few cases when called in consultation, when more than this time, by force of circumstances, had elapsed; and artificial delivery, either with or without the use of an anæsthetic, was proceeded with at once with successful terminations,— one of these a cross spasmodic contraction of the whole uterus.

We believe there is nothing gained by delay, as has been done for days and weeks, but much lost. The adhesions seldom diminish. The patient, until the secundines are delivered, cannot repose; she is in a constant state of anxiety; there can be no real advance in recovery. The physician must hold other practice in abeyance to this case; he is harrassed by day and by night by an impending danger from hemorrhage or septicæmia, or both. There seems to be little prospect of gain by waiting. The uterus is in its nearest physiological condition at the time of the birth of the child; its inherent contractility of tissue is then alive to every exciting cause. If there be inertia present, the necessary manipulations will excite and draw the forces of the system to the part and overcome it, while the longer the delay the more tolerant is the womb to the presence of a foreign body; so to speak, it accepts its partial relief by the birth of the child from over distention with satisfaction, and rests itself, unwilling to be disturbed.

We do not intend to pursue the subject of procedure in retained placenta, at term, in this paper to any extent. You may criticise the writer without an extended experience for presenting it, which is just the reason (want of experience) why it is presented. If any one with an extended personal knowledge and practice in cases of retained placenta can point out a better way than to avoid having them, it will be interesting information.

But there is another class of cases of retained placenta, a class of which doubtless we all have had more experience and annoyance than desired, and which might more properly come under another title — treatment of abortion — which we choose to bring under consideration at this time. Unfortunately, the rules for securing the expulsion of the placenta at full term or period of viability do not come materially to our aid here.

It is known that in abortion prior to the close of the second month of gestation the fœtus and secundines are usually expelled together, and if not, the latter are so small as ordinarily not to be a source of much danger even if not interfered with, but subsequent to that time until viability is a period causing the physician a deal of trouble. The placenta compared with the fœtus is proportionally of large size and of close attachment to the uterus, and inclined to adhere, as naively expressed by one of our patrons in a retention case of his wife: "I tell you, doctor, the thing isn't *ripe* yet."

It is rather the rule than otherwise, at this period, that cases are watched and time given them, but both patient and physician are filled with anxiety. There are oft-recurring and dangerous hemorrhages. There are bad odors, sometimes septicæmia, and always danger of it. Seldom is a case permitted to continue without some of these untoward features, while the treatment is apt to be confined to such efforts to obtain the placenta as can be achieved by one or two fingers, and ergot, which seldom succeeds in either bringing away the after-birth or materially checking hemorrhage, and, so failing, further measures are confined to the use of deodorizers, antiseptics, and the tampon is compelled to it, until nature, seemingly disgusted. with itself, if not with the doctor, rouses at last and expels the foul offender, or, failing, it passes away in sickening discharges, or possibly it is rarely absorbed.

The writer confesses these delays too much the habit of his own practice in earlier professional life, but of late years I rarely wait a quarter of a day, and often not an hour. Having of late years a business co-partner, hence a colleague easily at hand as an aid, I now proceed at once to remove the placenta artificially. In doing so, administer an anæsthetic, carry the hand into the vagina, remove the placenta. The results have been satisfactory.

Gentlemen, the manner of a majority of these cases coming to us for aid is known too well. The result of criminal practices on the part of parents even in wedlock, or of unfortunate girls under instruction or by the hand of professed abortionists, with the advice that when in trouble call upon a good physician. We some-[illegible]mes question the responsibility of the physician to respond to [illegible]uch cases, but often we are brought to the bedside unconscious of the nature of the case until the moment is imminent. Quicker than can the question arise the doctor's sense of duty to human life has decided his action. The question with us here is not what cases it is our duty to attend ; but how to do our duty well.

In the resume of our subject concerning the third stage of labor, we would recommend ordinarily (1) that efforts to deliver the placenta should be made within the first ten minutes following the close of the second stage, believing the vigorous uterus more responsive then than at a later period.

2d. That the application of the binder prior to the delivery of the placenta, except in inertia of the womb, and delay when the accoucheur could not remain by the side of his patient, is not only useless, but a hinderance.

3d. That neither mode, of traction alone or expression alone, is the most efficient or desirable means of aiding in the delivery of the after-birth, but if either mode alone is to be adhered to, that of expression has the advantage over traction in being the more efficient in completing this stage of labor, and also in preventing hemorrhage.

4th. That the best method known is the proper combination of the two methods (traction and compression) combined, keeping in remembrance that there is a vast difference between pressing the uterus and compressing or grasping.

5th. Never neglect maintaining the firm grasp of the uterus for a period of some minutes following the descent of the placenta.

6th. That the best treatment of retained placenta is to not have it — but, having it, to proceed with artificial delivery as soon as practicable ; better use the most efficient means that can be deemed judicious than to leave the patient with the greater risks of hemorrhage, and a subject of adynamic and ataxic fever.

7th. The best treatment of retained placenta in abortion is to "go for it" as promptly as possible.

DISCUSSION.

Dr. C. W. Brown: I have been much interested in the doctor's paper, and will take up but a very short time. I agree with his methods recommended for the delivery of the placenta, mine being about the same, with an exception that I will mention. As regards the time, I usually, as soon as the child is taken care of, proceed to deliver the placenta without regard to time. I place the left hand over the fundus of the uterus, grasping it as has been described, making a gentle kneading motion, and pressing as much as I am able to on the fundus of the womb. Instead of grasping the cord, my custom has been to follow up the cord, using it as a guide to the nearest edge of the placenta, and then to gradually work under the edge of it to excite contraction of the womb. I wait a reasonable length of time for the natural uterine contraction, and then, when that occurs, use the pressure on the outside and the fingers near the nearest edge of the placenta, and as the womb contracts and the placenta begins to come down I roll it on itself, so that it comes edgewise. Instead of pulling at the center of the cord, like an umbrella towards the wind, I bring it down rolled up, without reference to the position of the cord. I believe that this is an important thing, and my experience has been that I have had no trouble whatever in delivering the placenta in a large number of cases.

Dr. Elder: I have thought of the matter of delivering the placenta, and I have never had any trouble by retained placenta. There is one thing I have never done, used a forcible inspiration to fill the lungs with air. But I have tried to use forcible pressure. As to winding the cord about the hand and following it up, I have found it to result very satisfactorily.

Dr. Jewett: What has been said is so nearly sound, that retraction would be out of place. I hardly agree with placing a ligature upon the placental extremity of the cord. It would seem to me that it would prevent the placenta from disgorging; it is more bulky and difficult to remove. It seems to me that there is always a great advantage to allow the blood to escape, all that possibly could, from the placenta; you have less to remove, and I think it passes easier. I have never, except in a case where I knew there was a second child, applied a ligature to the placental extremity of the cord.

Dr. Johnson: In addition to the method of removing the placenta in what is called "unbuttoning the placenta," I always, about at the end of twenty minutes before the birth of the child, as near as my experience would allow me to guess at it, administered a dose of ergot to secure the uterine contraction and avoid uterine inertia. I think I have helped the patients in that way and promoted the third stage of labor. I do not know whether it is customary with others or not, but that has been my custom almost invariably. And in my experience of twenty-five or thirty years I have never had a case of retained placenta where it has been retained over twenty minutes or half an hour.

Dr. Hendrick: I deem it perfectly safe always to tie the cord, and sometimes an advantage. Usually it makes no difference. More generally the placenta is detached in the last effort of the uterus, and lies in the uterus; and, if so, disgorgement has already taken place; if not, the tying of the placental end of the cord serves to retain the blood, it is neater so far as the bed is concerned; and it is an acknowledged mode of aiding in the separation of the uterus if there is anything like adhesion; and that is the reason why I would do it. As to the giving of ergot twenty minutes before the expiration of labor and the birth of the child, I would not do it. I would be afraid of retained placenta, if given so early as that. Twenty minutes before the expulsion of the child is a very uncertain period. There may be many long weary hours before the child is born. That sudden action of the uterus is apt to throw it into a spasmodic contraction on the whole body, or an unequal contraction, which is one of the means of causing retained placenta.—*Medical Times*.

**Transfusion and Infusion.**—Dr. A. Landerer (*Virchow's Archiv*, August 3, 1886) discusses at length the present state of knowledge in the matter of the treatment of blood losses jeopardizing life, profound anæmia from other causes, and certain forms of poisoning. His conclusions are based upon the now well recognized dangers attending the transfusion either of blood or of serum, the inadequacy of salt solutions, and the efficiency of a mixed solution of salt and sugar. His experimental investigations in regard to the latter solution were conducted in the Physiological Laboratory in Leipsic, under the supervision of Prof. Ludwig and Dr. Gaule.

The dangers attending the transfusion of blood are illustrated by two cases observed by the author in the surgical clinic. The first of these was that of an eight year-old boy reduced to the last degree of anæmia in consequence of paralysis of the muscles of deglutition following diptheria. The details of the operation were carried out with the utmost care in accordance with the accepted rules of procedure. After thirty cubic centimetres of thoroughly defibrinated and filtered blood had been thrown into the left median vein, gasping respiration and facial spasm were followed by sudden death. At the post-mortem examination the pulmonary artery was filled with fresh clots.

The second case was that of a young woman suffering with acute anæmia. Here peritoneal transfusion (Ponfick) was performed with full antiseptic precautions, three hundred cubic centimetres of blood being introduced. The patient died of foudroyant peritonitis in forty-eight hours.

The investigations of Alexander Schmidt, Cramer, Edelberg and others have shown that these accidents are due to the development in the drawn blood of fibrinferment, which despite the most careful defibrination is occasionally the cause of fatal change in the blood of the organism into which it is introduced. That this change only occasionally occurs does not militate against the objections to transfusion, because in any given instance it can neither be foreseen nor prevented.

Since Goltz has shown that the immediate cause of death in blood loss is not, as formerly thought, dependent upon loss of hæmoglobin and its result, the deprivation ot oxygen, but rather that it is dependent upon certain mechanical conditions, especially a derangement of the blood-vessels and their contents and incomplete filling of the heart with blood, the introduction of some indifferent fluid not injurious to the blood itself, in order to fill the vascular system, became the controlling indication.

For this purpose the 0.6 per cent. alkaline solution ot common salt (Kronecker and Sander, *Berlin. Klin. Wochenschr*, 1879, No. 52) has been regarded as possessing the necessary qualities. In fact, as Von Ott demonstrated, animals thus treated after severe bleedings make a better convalescence and repair the loss of red corpuscles and other bloody constituents more rapidly than those in which transfusion with whole or defibrinated blood has been performed.

Furthermore, the transfusion of blood was, even under the most favorable circumstances, followed in almost all cases by fever of longer or shorter duration, usually ushered in by rigors, great mental and physical depression, præcordial distress, frequently by intestinal disturbance, and occasionally by exanthems — symptoms of the "fibrin-ferment intoxication" of Edelberg.

Efforts to prevent the excessive formation of fibrin-ferments by changes in the methods of defibrinating the blood, as by agitating it with broken charcoal, glass beads, etc., have been without result, nor have attempts to prevent clotting by the admixture of the blood with other fluids led to any practical result.

The author regards with disfavor attempts to prevent coagulation by any admixture of peptone.

A very simple method, yet one not wholly free from objection, consists in a combination of blood transfusion with the infusion of the solution of salt. This expedient meets one of the objections to the transfusion of blood: namely, that the volume of fluid introduced is frequently too small to correct the mechanical disturbance between the capacity of the vessels and their contents. The admixture of two to three hundred cubic centimetres of blood with seven to eight hundred cubic centimetres of 0.6 per cent. salt solution affords sufficient fluid on the one hand and reduces the danger of poisoning by fibrin-ferments. This method of "mixed transfusion" will probably be preferred as less dangerous by those who cannot abandon the view that transfusion is the rational procedure.

The use of various forms of serum is not without risk, seeing that they all, with the single exception of horse serum, contain more or less ferment. Serum is not practicable for the further reason that its preparation is too difficult and tedious.

Other fluids containing nutritive substances, as for example milk, have been found to be attended by direct dangers.

Much can also be said against the infusion of salt solution. In cases of poisoning in which transfusion is required, salt solutions would be clearly inadequate. In fact, the use to replace lost blood of a fluid which is scarcely more than water, without value in nutrition or force, must be looked upon as at best an unsatisfactory and imperfect procedure. This objection — that such a solution acts merely mechanically — is a serious one. In the words of Landerer, "it is as if one were to offer a hungry man a stone instead of bread." Furthermore, it has not been followed in a large number of cases, especially where there has been large blood loss, with the hoped-for result. Nevertheless, we have in salt solution probably hit upon a basis by means of which, with proper additions and modifications, a transfusion fluid meeting the various indications for transfusion under all circumstances may be made up.

The requirements of such a fluid are as follows:

1. It must be absolutely without danger in itself.

2. It must be readily obtainable under all circumstances, so that in case of necessity it may be procured at once.

3. It must contain the greatest available amount of assimilable substances capable of sustaining animal heat and the powers of the organism.

4. It should be of the simplest possible chemical constitution.

The author believes that in a combination of the alkaline salt solution (0.6 per cent.) with a solution of sugar (one to three per cent.) we have a fluid which fulfills all these requirements.

The conclusions of the author are as follows:

1. Blood transfusion is dangerous and useless.

2. The infusion of a solution of common salt does not in really severe blood loss (more than four and a half per cent.) by any means insure a favorable result.

3. A combined solution of salt and sugar is preferable, because (*a*) it favors and accelerates the natural tendency of the organism to readjustment by favoring the transudation of fluid from the tissues to the interior of the blood vessels; (*b*) it affords the organism a light and quickly assimilable nutrient material; (*c*) it more closely resembles the blood in density than the solution of common salt; (*d*) it raises the blood pressure.

4. The combined solution of salt and sugar may be of use in poisoning by nitro-benzol, chloral hydrate, chloroform, carbon dioxide, etc.

5. In cases of loss of the watery portions of the blood alone, as in cholera, the salt solution may be more available than the combined salt and sugar solution.—*Dr. J. C. Wilson in Med. Times.*

**Acute Otorrhœa in Children.**—Dr. G. C. Pardee, in the *Pacific Med. and Surg Journal*, says that all that is needed in the way of instruments are an ordinary half-ounce rubber syringe, a little piece of wire, such as a straightened hairpin, and some absorbent cotton. Armed with these simple and inexpensive instruments, a few ounces of a one-per cent. solution of carbolic acid, and a little finely powdered boracic acid, let the practitioner proceed as follows: Let him gently wash out the affected ear with the syringe and the carbolized water, warmed, using three or four syringefuls. Then let him have the nurse take the little patient to the window and allow the sunlight to fall directly into the affected ear, while he carefully and gently dries the canal with a bit of cotton wrapped around the roughened end of the hairpin probe, straightening the canal for this purpose by drawing the concha upward and backward. Then let him have the child placed on its side with the affected ear upward, and let him fill the canal nearly full of the powdered boracic acid, plugging the meatus finally with a bit of cotton. Let him repeat this process a few times at intervals of twenty-four hours, and he will be surprised to find how quickly a recent discharge will cease, and the ear regain its healthy condition. If after a week or ten days' trial he finds, as he seldom will, that the discharge does not decrease in quantity, let him throw aside the "dry treatment" and try the "wet treatment," beginning with a weak solution of nitrate of silver — say five grains to the ounce — gradually increasing the strength if the discharge does not yield. In all cases and under all circumstances, however, he should not forget that here, more than anywhere else, "cleanliness is next to godliness," and that frequent syringing with a warm antiseptic solution is the only way to keep the stagnating and decomposing secretions from irritating the mucous membrane and perpetuating the discharge.—*Epitome.*

**Delirium Tremens.**—Dr. J. T. Whittaker (*Cincinnati Lancet-Clinic*) says that signs of delirium tremens rarely appear until derangement of digestion is noticed, and the history of most cases shows a gastric catarrh which prevents the absorption of the remedies. Gastric catarrh produces delirium tremens by impairing nutrition. The secret of success in the treatment is to feed the patient properly. A man may drink and continue to drink, and show no delirium tremens. But as soon as vomiting begins, with the other pronounced symptoms of gastric catarrh, the mind begins to waver and the whole nervous system to flag.

But not every drinker suffers delirium tremens. Alcohol is a food in the amount of one gramme to a kilo of weight (fifteen grains to two pounds) in twenty-four hours- The speaker frequently has had occasion to testify as to the testamentary capacity of individuals addicted to alcohol, and has familiarized himself with the subject. Eight grammes to a kilo will kill, and one hundred grammes will produce disease and true delirium tremens in some cases, pretty much always, with the prelude of a gastric catarrh. So the tongue is coated, the breath foul, the bowels constipated, as a rule, throughout the disease.

The secret of success is found when food is given in connection with the treatment of the cases. But the question is how to get food into the body. The gastric catarrh produces vomiting and thereby the food is rejected. If this be the case it is highly important to inject the food into the bowel. Thus the patient is easily nourished by chopped beef and pancreas. To feed the exhausted nervous system is the best way to give it rest.

He had seen many cases of delirum tremens, but never one which required the administration of chloroform. He would, however, not hesitate to resort to it if the necessity arose. As a rule, its use is unjustifiable. He would not hesitate to give bromide of potassium in ℥ ss doses if necessary to secure sleep. He does not consider it more dangerous than common salt. He gives drachm doses every four. hours, largely diluted, of course, in hypochondriasis and hysteria.

There is no better food by the mouth than milk. If this be rejected, then recourse must be had to rectal alimentation. The vomiting can be controlled by a dose or two of the officinal morphia solution (U. S. P.) with a drop or two of dilute hydrocyanic acid.

The quickest and best results are obtained by abstention from all anodynes, except, perhaps, the bromides. Steer away from anodynes, but secure sleep by feeding the nervous system. In convalescence nothing is better than hydrochloric acid, five to seven drops, largely diluted before meals.—*The Epitome.*

**Menthol.**—Under the names of peppermint, stearescence, stearoptene of peppermint, and peppermint camphor, menthol has been known and described since the latter part of the previous century; and tradition has it that to the Japanese and Chinese this substance has been known for centuries. * * * Early in 1880 a large consignment of menthol reached New York, and as the drug rapidly gained in popularity, the shipments since received have been enormous. In 1881 Dundas, Dick & Co. introduced the now famous cone to successful use in this country, in which enterprise he was closely followed by other houses, until to-day there may be found some thirty makers of cones of diverse grades of purity. A pure menthol should give a clear, colorless fluid when raised to about the boiling point of water. A yellow, pink or blue colored menthol should be rejected, as the cones made therefrom will increase in unsightliness with age, The chief adulterants of the menthol cones of the day are paraffin, salicin, stearic acid and white wax. The cones manufactured by reliable houses being, as a rule, pure.—*S. Casket, in the Druggist Circular, June, 1886.*

**Cascara Cordial.**—Touching the properties of Cascara Cordial as prepared by Parke, Davis & Co., we extract the following from a paper read before the Medical and Surgical Society of the Kanawha Valley, September 1, 1886, by R. S. Henry, A.M., M.D., Charleston, West Va. In addition to its power of disguising the taste of such bitter drugs as qunine, its gentle laxative properties render it peculiarly well adapted for addition as a corrigent to the many preparations which, given alone for any length of time, tend to interfere with the normal action of the bowels, such as the various preparations of iron and opium, than which no others are more frequently indicated and more used by physicians.

Take, for example, such a preparation as the tincture of the chloride of iron. What physician has not been compelled to desist from its use, even when most strongly indicated on account of the marked constipation it causes? To obviate this objectionable feature of this valuable hæmatic, we may use the following formula, or such modifications of it as may be desirable:

℞. Tinct. ferri chlor., ........................ ʒ vj to ʒ xij,

Cascara cordial ........................ ʒ ij,

Water, q. s. ad ........................ ℥ iv.

M. Sig.—Teaspoonful, T. I. D.

A very useful formula in the treatment of the anæmia and glandular enlargement of pale, flabby, scrofulous children, is the syrup of the iodide of iron combined with cascara cordial, as fol-

℞. Syrup ferri iod., } aa ........................ ℥ ij.

Cascara cordial, }

Dose—20 drops in water, T. I. D., increased as required.

Perhaps no drug is more universally prescribed than quinine. When it is desired to produce an immediate absorption of this drug, it is preferable to give it in the form of powder or solution. Its inherent bitterness, however, renders this mode of administration objectionable to most patients. Innumerable vehicles have been tried to disguise its bitterness, with varying success, but we believe none has proven so acceptable as cascara cordial, which has been extensively used for this purpose, and with most gratifying results. To abort an ague fit the following may be prescribed with advantage:

℞. Quiniæ sulphat..............................grs. x,

Cascara cordial.... ............................ʒ ij,

Water.............................................℥ j.

M. S.—Take at a draught.

As a prophylactic against malaria, or in all malarial remittent affections, we may combine quinine with cascara cordial as follows:

℞. Quiniæ..........................................ʒ ij,

Cascara cordial.................................℥ ij,

Water, q. s. ad.................................℥ iv.

M. S.—Teaspoonful three or four times a day; increasing the quinine when indicated.

When it is desired to give the preparations of opium and to antagonise their constipating effect the addition of cascara cordial to the dose of the narcotic will admirably meet the indications. Thus, when it is required to use opium for its abortive effect in the initiatory stages of incipient catarrh of the respiratory tract in the first stages of a cold, in the head or cough, we may give:

℞. Dover's powder...........................grs. x,

Cascara cordial..............................ʒ ij,

Water.........................................℥ j.

M. Sig.—At a draught.

The cascara here secures a satisfactory evacuation of the bowel, and thus has a derivative effect on the catarrhal condition, which is further aided by the well-known anti-phlogistic action of Dover's powder.

It is often difficult, when necessary to give opium in some form for a long time, to counteract its repressant action on the secretions, especially on those of the intestines. It not only secures a free painless stool, but acts also as a stomachic, improving the process of assimilation, which are is often at fault in cases of chronic invalidism.

A great objection to some very valuable remedies consists in the disagreeable cerebral symptoms accompanying their administration in large doses, or in cases in which it is necessary to prolong their use for a considerable period. Thus salicylate of sodium, which is so invaluable in rheumatism, must often be stopped at a critical period in the process of the disease, or the dose reduced, on account of the head symptoms developed; cascara cordial will often prevent the development of these toxic symptoms of the drug. The following furnishes a convenient formula for combining the salicylate with cascara cordial:

℞. Sodii salicylat...................................℥ j,
Cascara cordial................................℥ ij,
Water, q. s. ad..................................℥ iv.

M. Sig.—Teaspoonful in a wineglass of water four times a daily, increasing or diminishing the salicylate as required.

The few illustrations given imperfectly, convey the very wide range of application of cascara cordial as a vehicle. The requirements of the physician will extend its use to almost all prescriptions needing correction, either on account of their bitterness or tendency to constipate. So many diseases are dependent upon or attended by disturbance of the functions of digestion and assimilation, and especially by interference with the secretory functions of the bowels, and so many valuable drugs, the use of which is indispensable, but serve to increase or perpetuate this tendency, that the application of a vehicle which secures palatableness and at the same time establishes a regular action of the bowels, must come into very general use in the every-day routine practice of a physician.

**Treatment of Offensive Diarrhœa in Children.**—There is a form of diarrhœa in children, usually occurring after weaning, and from that period to four or five years of age, which is characterized by the most horrible offensiveness of the motions. This is so marked that it is generally at once mentioned by the parents. It is commonly met with in summer, but is not strictly what is known as infantile diarrhœa; in this disease the stools are sour, but not necessarily fœtid. Probably this form of diarrhœa differs from the diarrhœa of younger infants in being caused by the growth of the ordinary bacteria of putrefaction. It is not amenable to treatment by an astrigent, nor has any alteration f o diet much effect upon it.

Dr. James Braithwaite, *British Medical Journal*, however, thinks that it may be successfully treated by disinfecting the bowel contents by means of salicylate of iron, as in the following prescription, which is suitable for a child of two years of age:

℞. Sulphate of iron.................................℈ i,

Sulphate of sodium.................................℈ i,

Glycerin.................................ʒ iij,

Water.................................℥ iij.

The iron and the salicylate should be dissolved separately, and the solutions mixed. The color is darker than port wine. and the taste not unpleasant. One teaspoonful must be given every hour, until the stools become blackened, which happens in about twenty-four hours; or a larger dose may be administered at longer intervals. The medicine should then be given every three or four hours, and occasionally a small dose of castor oil, to clear the bowels well out, and to get the secondary constipating effect of the oil.

In hospital practice, and amongst the poor, it is not so successful as it would be if it were possible to remove the child from the family living room, the air of which is usually very impure, and is made worse by the smells incidental to cooking, and the presence of a sink.—*Therapeutic Gazette.*

**Constipation.**—After the reading of a paper on this subject by Dr. Arthur V. Meigs, at the meeting of the College of Physicians, May 5, 1886, Prof. Da Costa made the following interesting remarks :

A point of particular interest is the occurrence of fever in these cases. I saw the fourth case with Dr. Meigs, and the fact that it simulated typhoid fever so closely is a matter of interest. I have seen a similar case in which there were almost identical symptoms, with an almost identical termination. We see from this case and the others referred to, that constipation may cause fever which is continued and may present the symptoms of a low type.

There is another point connected with the occurrence of constipation in fever to which he did not have occasion to allude; that is to say, sometimes after low fevers in which the state of constipation to which he has called attention occurs, relapse of the fever will be developed by the constipation. We grope around in darkness, wondering what may be the cause, thinking that it is a true typhoid fever relapse, when by giving small doses of oil or of laxatives, both the fever will disappear and the bowels be frequently moved. I have seen this state of things keep up for five or six days; and I think that a good many cases of relapse in typhoid fever have their origin in the very condition to which Dr. Meigs has alluded and to which I now call the attention of the College.

I will go further; I have reason to think that in some of these cases there may be well-developed typhoid fever symptoms with rash, due to constipation, which will disappear when the bowels are moved. I have seen the same thing after remittent fever. It seems to me that the occurrence of constipation after fever, typhoid or malarial, may lead to the re-development of the febrile state, which may be considered a relapse, when in reality it is only the same kind of irritation of the bowels, as in the case cited by Dr. Meigs, producing a fever of low type, when there was not the slightest reason for suspecting typhoid fever.—*American Medical Digest.*

**Antiseptic Dressing in Amputation.**—The following remarks, on a case by Dr. Thomas G. Morton, before the Pennsylvania Hospital (which we find in the Philadelphia Medical Times), will serve to illustrate the antiseptic method: A patient, a man twenty years of age, was then etherized. After the leg was washed with soap, shaved, and cleansed with bichloride solution, an Esmarch bandage (previously disinfected) was placed on the limb. The field of operation was next uncovered and surrounded with towels wet with the same solution. The knives were kept in the tray immersed in a 3-per cent. solution of carbolic acid. The circumference of the limb was taken at the place where it was intended to divide the bones; the long flap was then mapped out, rectangular in shape, one-half the circumference of the limb; the short flap was one-fourth the length of the long flap. The arteries were then tied with carbolized catgut, and the wound carefully cleansed of all spiculæ of bone or blood-clots; a few catgut threads were introduced as a drain, the flaps brought together with interrupted sutures of catgut, the protective placed over the sutures, and iodoform and bichloride gauze was applied. The whole stump was finally covered with dry absorbent cotton (previously treated with a solution of bichloride), kept in place with a wet gauze bandage similarly treated with the mercurial solution. The patient was then covered up warm and a bottle of hot water placed to the remaining foot. These dressings will remain undisturbed for three weeks or longer, unless some unexpected symptoms appear calling for earlier interference.

**Ingluvin.**— Ingluvin is prepared by the far-seeing chemists, Wm. R. Warner & Co., of Philadelphia. It is made from selected gizzards, and is so carefully extracted as to be free from all foreign organic bodies. It is already known and appreciated by the medical profession. The *American Analyst* bespeaks for it the same appreciation by its readers. We extract the following:

Prof. Roberts Bartholow, M. A., M. D., LL. D., in his late work on "Materia Medica and Therapeutics," says:—INGLUVIN. This is a preparation from the gizzard of the domestic chicken—*ventriculus callosus gallinaceus.* Dose, gr. v.—℈ j.

Ingluvin has the remarkable property of arresting certain kinds of vomiting — notably the *vomiting of pregnancy.* It is a stomachic tonic, and relieves indigestion, flatulence and dyspepsia.

The author's experience is confirmatory of the statements which have been put forth regarding the exceptional power of this agent to arrest the vomiting of pregnancy. It can be administered in inflammatory conditions of the mucous membrane, as it has no irritant effect. Under ordinary circumstances, and when the object of its administration is to promote the digestive function, it should be administered after meals. When the object is to arrest the vomiting of pregnancy, it should be given before meals.—*From the American Analyst, August 1, 1886.*

**Sleeplessness.**— Dr. J. Miller Fothergill says of sleeplessness: "One broad rule to bear in mind is this: Opium is the agent where insomnia is due to pain ; chloral where it is due to a high blood pressure in the arterial system ; the bromides where there is any peripheral irritation. Opium, having a pronounced effect upon the sensory portion of the brain is an analgesic, is the drug par excellence in sleeplessness due to pain. Whenever there is a morbid condition in tense tissues, as syphilitic node for instance, pain on going off to sleep is set up by that dilation of the system generally which is essential to brain depletion. The effect of pain is to arouse the brain into wakefulness. Where such a complication exists, it is well to combine the opiate with some potent depressent of the circulation, as antimony or aconite. In many cases a full dose of alcohol is sufficient for the attainment of the desired end."—*Brief.*

**Vinegar as an Antiseptic in the Treatment of Diphtheria.**— Dr. Engelmaan (*Centralblatt fur Klin. Med.*, No. 14) uses vinegar, either the officinal dilute acetic acid or ordinary cider vinegar, diluted with an equal quantity of water as a gargle, or undiluted applied upon a brush. It is also used in spray diluted once or twice. It is antiseptic, not caustic, and has a more agreeable taste than many of the antiseptics usually employed.—*Medical Times.*

